# Association between muscle mass and diabetes prevalence independent of body fat distribution in adults under 50 years old

**DOI:** 10.1038/s41387-022-00204-4

**Published:** 2022-05-28

**Authors:** Melanie S. Haines, Aaron Leong, Bianca C. Porneala, James B. Meigs, Karen K. Miller

**Affiliations:** 1 Neuroendocrine Unit, Massachusetts General Hospital, Boston, MA USA; 2 Harvard Medical School, Boston, MA USA; 3 Division of General Internal Medicine, Massachusetts General Hospital, Boston, MA USA

**Keywords:** Diabetes, Epidemiology

## Abstract

**Background/objectives:**

Although relatively less muscle mass has been associated with greater diabetes prevalence, whether there is an association between muscle mass and diabetes prevalence independent of body fat distribution is unknown. The objective was to determine whether less skeletal muscle mass is associated with greater diabetes prevalence in young men and women independent of body fat distribution.

**Subjects/methods:**

One thousand seven hundred and sixty-four adults, aged 20–49 years old, from the United States National Health and Nutrition Examination Survey (2005–2006). Body composition, including appendicular lean mass (ALM), was measured by dual-energy x-ray absorptiometry. Diabetes was defined as fasting blood glucose ≥7 mmol/l, 2-h blood glucose ≥11.1 mmol/l on 75 g OGTT, HbA1c ≥ 48 mmol/mol (6.5%), use of diabetes medications, or self-reported diagnosis of diabetes.

**Results:**

The odds of diabetes were 1.31 times higher in men [OR 1.31 (1.18–1.45), *p* = 0.0001], and 1.24 times higher in women [OR 1.24 (1.05–1.46), *p* = 0.01], per percent decrease in ALM/weight after controlling for age, race, height, smoking, and education. After additionally controlling for android/gynoid fat, the odds of diabetes were 1.20 times higher per percent decrease in ALM/weight in men [OR 1.20 (1.04–1.37), *p* = 0.01]; an inverse association was also observed in women, albeit was not statistically significant [OR 1.08 (0.90–1.30), *p* = 0.42].

**Conclusions:**

Less muscle mass was associated with greater diabetes prevalence independent of body fat distribution in young men. The association was not statistically significant in women after controlling for android and gynoid adiposity. Low muscle mass could be a causal factor in the development of type 2 diabetes or a correlated marker of higher metabolic risk.

## Introduction

Although obesity is a strong risk factor for type 2 diabetes, there is significant variation in the prevalence of type 2 diabetes among adults of comparable body mass index (BMI), demonstrating the importance of risk factors other than overall body weight [1, 2]. This variable risk is partially attributable to differential adipose depots, with trunk and upper body (i.e., android) fat being more pro-inflammatory and having a more adverse cardiometabolic effect than gluteofemoral (i.e., gynoid) fat [3]. However, adipose depots alone do not fully explain differential type 2 diabetes risk. Considering that skeletal muscle is the most insulin-sensitive tissue and responsible for 70–90% of post-prandial glucose disposal [4], lower skeletal muscle mass relative to adiposity may be an important marker of type 2 diabetes risk.

Studies using data from the United States National Health and Nutrition Examination Survey (NHANES) have demonstrated that lower appendicular lean mass (ALM)/BMI is associated with greater insulin resistance in older adults [5], lower skeletal muscle index [the ratio of skeletal muscle mass (estimated by bioelectrical impedance) to total body weight] is associated with both insulin resistance and diabetes in adults younger and older than 60 years [6], and percent lean mass is negatively associated with HbA1c in both men and women without diabetes, and in men <40 years old with diabetes [7]. However, none of these studies controlled for fat deposition patterns that vary by sex and are also associated with type 2 diabetes risk, and only one of the studies performed sex-stratified analyses [7], which are critical given known sex differences in muscle and type 2 diabetes risk. In addition, data in older adults cannot be extrapolated to younger adults (i.e., under 50 years) because younger adults have not experienced age-related declines in muscle mass and generally develop type 2 diabetes at a higher BMI than older adults [8]. Finally, whether there are race-specific differences in the association between skeletal muscle mass and diabetes prevalence in young American adults is unknown. Understanding whether there are sex and race differences in the association between muscle mass and type 2 diabetes may yield important insights into known sex and race disparities in type 2 diabetes.

We hypothesized that less skeletal muscle mass is associated with greater diabetes prevalence in both young men and women in the United States, independent of body fat distribution. In a secondary analysis, we sought to determine whether less skeletal muscle is associated with greater diabetes prevalence independent of body fat distribution in Hispanics vs non-Hispanic Blacks vs non-Hispanic Whites.

## Materials/subjects and methods

We analyzed cross-sectional data from the 2005–2006 NHANES, as that was the most recent year with body composition data. The survey uses a multistage, complex, stratified probability sampling design that oversamples minorities and is representative of non-institutionalized adults in the United States, providing excellent external generalizability. The survey has been conducted and managed by the Centers for Disease Control and Prevention since 1971, and its contents and procedure manuals are available online at http://www.cdc.gov/nchs/nhanes/htm. This study was exempt from local Institutional Review Board review due to the de-identified nature of the data analyzed.

We restricted our sample to individuals aged 20–49 years old. We excluded subjects who were pregnant, nursing, or status-post bilateral oophorectomy given changes in body weight, body composition, endocrine hormones, and/or type 2 diabetes risk in these groups. We also excluded subjects who were prescribed testosterone, growth hormone, or glucocorticoids given the known effects of these endocrine hormone deficiencies and/or their replacement on body composition and/or type 2 diabetes risk. Individuals whose height was >192.5 cm and/or whose weight was >136.4 kg were excluded due to limitations of the DXA table. Of 10 348 participants in NHANES 2005–2006, 1764 eligible participants were included in this analysis.

### Body composition variables

All body composition measures were assessed using a DXA QDR-4500 Hologic scanner (Bedford, MA). Android and gynoid regions were defined by the Hologic APEX software used in the scan analysis. The android region is the area around the waist between the mid-point of the lumbar spine and the top of the pelvis; the gynoid area lies between the head of the femur and mid-thigh. Appendicular lean mass (ALM) was defined as the sum of the muscle mass of both legs and arms.

### Covariates

The following data were self-reported using questionnaires and included in analyses because they have been independently associated with type 2 diabetes risk: age, sex, race/ethnicity [9, 10], education [11], smoking status [12], and physical inactivity [13]. Race/ethnicity was categorized as Hispanic (combining Mexican American and other Hispanic), non-Hispanic White, non-Hispanic Black, and other (including multi-racial). For race-stratified analyses, “other” was excluded. Education was categorized as (1) less than 12th grade, (2) high school graduate or General Education Diploma (GED) equivalent, or (3) higher. For race-stratified analyses, education was collapsed into two categories of (1) less than 12th grade or (2) high school graduate, GED equivalent, or higher. Smoking status was categorized as never smoker (smoked <100 cigarettes in life), former smoker (do not now smoke cigarettes), or current smoker (smoke cigarettes every day or some days). Individuals were defined as physically inactive if they reported no vigorous or moderate activity of at least 10 min over the past 30 days that caused light to heavy sweating or slight to large increases in breathing according to the standard NHANES physical activity questionnaire. This definition of physical inactivity was chosen because prospective studies in both women and men have demonstrated that *any* level of self-reported physical activity is associated with a lower risk of type 2 diabetes compared to no physical activity [14, 15]. In a secondary analysis, physical activity was coded according to whether the individual met the American Heart Association’s (AHA) recommendations for physical activity in adults (150 min of moderate-to-vigorous aerobic activity per week, or 75 min of vigorous aerobic activity per week) [16]. An electronic digital scale, calibrated in kilograms, was used to assess weight, and a stadiometer was used to measure height after a deep inhalation. BMI was calculated as weight in kilograms divided by height in meters squared. Height was included as a covariate in analyses given the linear relationship between height and muscle mass [17].

### Diabetes variables

All techniques in NHANES followed the guidelines put forth by the American Diabetes Association. A fasting glucose blood test was performed in the morning after a 9-hour fast; subsequently, a 75-g oral glucose tolerance test (OGTT) was performed. Exclusion criteria for oral glucose tolerance testing included hemophilia or chemotherapy safety exclusions, fasting <9 h, taking insulin or oral medications for diabetes, refusing phlebotomy, and not drinking the entire Trutol™ solution within the allotted time. Plasma glucose was measured using a hexokinase method (Roche/Hitachi 911), and samples were processed, stored, and shipped to Fairview Medical Center Laboratory at the University of Minnesota for analysis. Glycosylated hemoglobin (HbA1c) was measured using HPLC (Tosoh Medics, Inc., San Francisco, CA). HbA1c samples were processed, stored, and shipped to the Diabetes Laboratory at the University of Minnesota for analysis.

Diabetes was defined by the presence of one or more of the following conditions: (1) HbA1c ≥ 48 mmol/mol (6.5%); (2) fasting glucose ≥7 mmol/l (126 mg/dl); (3) a 2-h glucose on an OGTT of ≥11.1 mmol/liter (200 mg/dl); (4) self-reported diagnosis of diabetes; or (4) self-reported use of diabetes medications (oral hypoglycemic agents and/or insulin) as previously defined [18]. Because 73 subjects were missing HbA1c, fasting glucose, and 2-h glucose on OGTT, diabetes was defined as self-reported diagnosis or self-reported use of diabetes medications in a secondary analysis.

### Statistical analysis

All data were downloaded, merged according to NHANES guidelines, and analyzed incorporating sampling weights, primary sampling units, and strata as supplied by NHANES. Continuous variables are represented as mean ± SD and categorical variables as count (percent).

To determine sex-stratified differences in clinical characteristics between those with and without diabetes, we performed Wilcoxon tests to compare continuous variables, Chi-squared tests to compare categorical variables, and Fisher’s exact tests to compare categorical variables if count <10 in either cell.

To determine the association between percent ALM/weight (primary predictor) and diabetes prevalence (primary outcome), we performed a series of multivariate logistic regression models: (1) a sex-stratified model (primary hypothesis), (2) a sex-combined model that included an interaction term for percent ALM/weight with sex, and (3) a sex- and race-stratified model. We adjusted for demographic covariates (i.e., age, sex, race, smoking, and education) known to be associated with type 2 diabetes risk, height, and physical inactivity, as well as the ratio of android/gynoid fat because it varies by sex and because android fat is detrimental towards, whereas gynoid fat is protective against, type 2 diabetes. A power calculation for the primary endpoints was as follows: in a sample of 1764 participants, assuming alpha = 0.05, power = 0.8, and a diabetes prevalence of 5%, we could detect an odds ratio of 1.15 between ALM/weight below the median vs ALM/weight above the median. Data are presented as an odds ratio with 95% confidence interval and associated *p* value for each model.

We used SAS (version 9.2 or 9.3; SAS Institute, Cary, NC) for all analyses and applied procedures to account for NHANES 2005–2006 sampling probabilities and complex sampling design in all models. Multiple imputations was applied to address potential bias resulting from nonrandom missing DXA data [19]. Five complete data files that contained both the non-missing and imputed values (generated using sequential multivariate imputation) were created. A two-sided *p* value ≤0.05 was considered a statistically significant test of the hypothesis that less skeletal muscle mass is associated with greater diabetes prevalence independent of body fat distribution in both young men and young women in the United States.

## Results

### Clinical characteristics

The clinical characteristics of 958 men and 806 women in our study sample, by diabetes status, are listed in Table 1. Diabetes was present in 5.2% of men and 5.1% of women, consistent with the young age of this population. Individuals with diabetes were older, had higher BMI, lower ALM/weight, higher android/gynoid fat, and were more physically inactive than those without diabetes. Using AHA guidelines, 77% of women with diabetes (*n* = 27), 63% of women without diabetes (*n* = 293), 63% of men with diabetes (*n* = 26), and 55% of men without diabetes (*n* = 345) did not meet recommended physical activity guidelines. In men, there was a larger proportion of Hispanic adults, and a smaller proportion of non-Hispanic White adults, with diabetes than without diabetes. In women, there was a larger proportion of the less than 12th grade education category, and a smaller proportion of the more than 12th grade education category, among those with diabetes compared to those without diabetes. Correlations between ALM/weight and anthropometric or DXA-derived adiposity measures are provided in Table 2.

**Table 1:** Clinical characteristics (mean ± SD).

	Men		*p* value	Women		*p* value
	With diabetes	Without diabetes		With diabetes	Without diabetes	
Number, *n*	50	908		41	765	
Age, years	40.4 ± 7.1	34.1 ± 8.7	<0.0001	40.0 ± 6.6	34.1 ± 8.7	<0.0001
Race			0.008			0.130
Non-Hispanic Black, *n* (%)	11 (22%)	209 (23%)		12 (29%)	188 (25%)	
Non-Hispanic White, *n* (%)	14 (28%)	425 (47%)		13 (32%)	363 (48%)	
Hispanic, *n* (%)	25 (50%)	274 (30%)		16 (39%)	214 (28%)	
Body mass index, kg/m^2^	30.6 ± 6.1	27.6 ± 4.5	0.0002	32.6 ± 7.6	28.0 ± 6.5	<0.0001
Weight, kg	91.1 ± 19.5	85.3 ± 15.6	0.029	85.6 ± 21.7	73.9 ± 17.9	0.0002
Appendicular lean mass (ALM), kg	26.9 ± 5.0	27.1 ± 4.4	0.816	19.9 ± 4.0	18.2 ± 3.9	0.003
ALM/weight, %	29.8 ± 3.2	32.1 ± 3.1	<0.0001	23.7 ± 2.4	24.9 ± 2.9	0.008
Android/gynoid fat	0.8 ± 0.2	0.6 ± 0.2	<0.0001	0.6 ± 0.2	0.4 ± 0.1	<0.0001
Smoking status			0.640			0.742
Never smoker, *n* (%)	25 (50%)	457 (50%)		25 (61%)	483 (63%)	
Former smoker, *n* (%)	11 (22%)	157 (17%)		5 (12%)	113 (15%)	
Current smoker, *n* (%)	14 (28%)	294 (32%)		11 (27%)	169 (22%)	
Education			0.985			0.0004
Less than 12th grade, *n* (%)	14 (28%)	245 (27%)		18 (44%)	147 (19%)	
12th grade, *n* (%)	12 (24%)	225 (25%)		9 (22%)	163 (21%)	
More than 12th grade, *n* (%)	24 (48%)	438 (48%)		14 (34%)	455 (60%)	
Physically inactive, *n* (%)	22 (44%)	264 (29%)	0.020	21 (51%)	223 (29%)	0.003

**Table 2:** Correlation matrix of body composition variables.

Grey boxes signify correlations in men. White boxes signify correlations in women. All correlations are significant at *p* < 0.0001.

### Association between skeletal muscle mass and diabetes prevalence

**Fig. 1:**
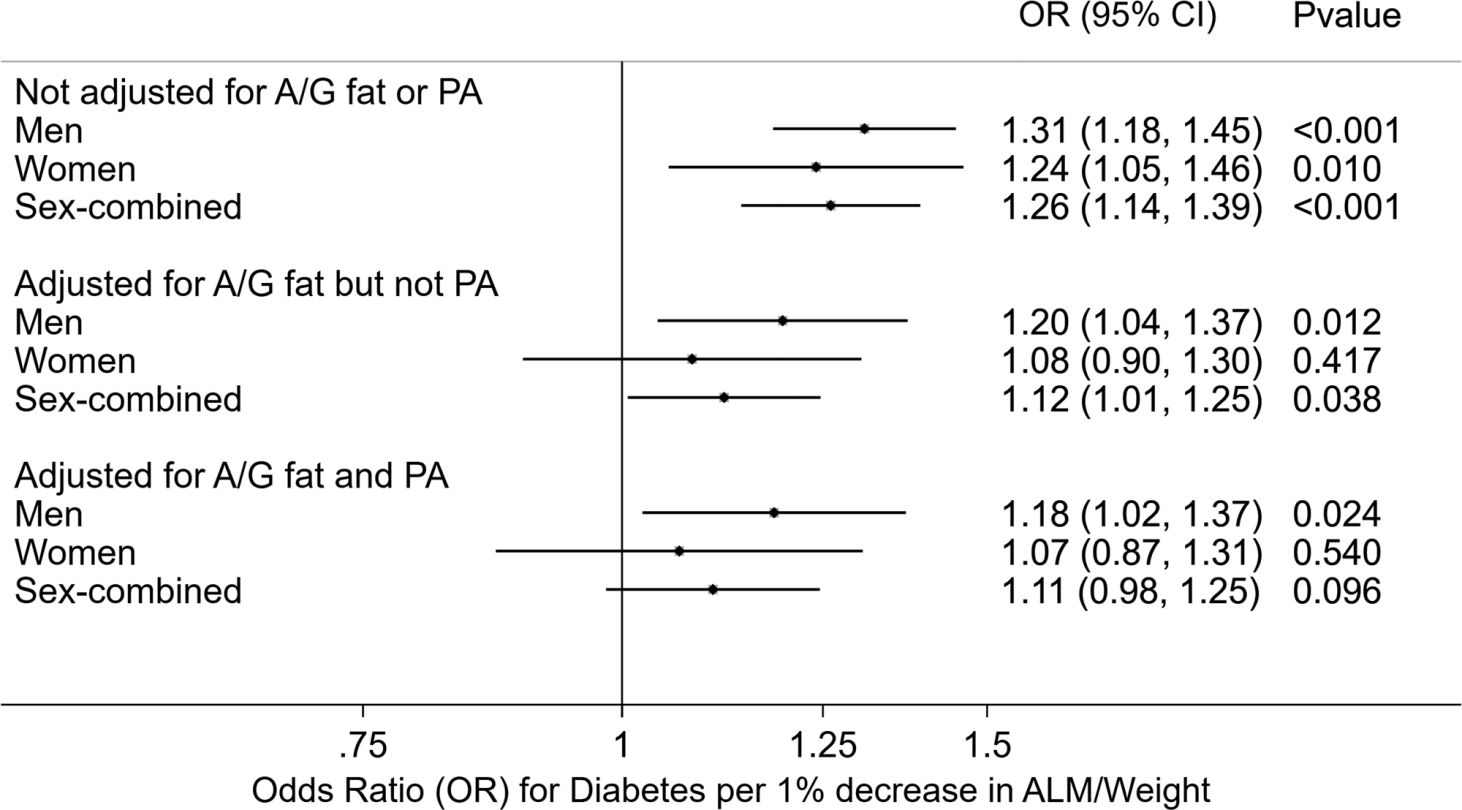
The odds of diabetes were 1.31 times higher in men and 1.24 times higher in women per percent decrease in appendicular lean mass (ALM)/weight after controlling for age, race, height, smoking, and education. After controlling for android/gynoid (A/G) fat, the odds of diabetes were 1.20 times higher for each percent decrease in ALM/weight in men an inverse association was also observed in women albeit was not statistically significant. After additionally controlling for physical inactivity (PA), the odds of diabetes were 1.18 times higher for each percent decrease in ALM/weight in men an inverse association was also observed in women albeit was not statistically significant.

The odds of diabetes were 1.31 times higher in men [OR 1.31 (1.18–1.45), *p* = 0.0001], and 1.24 times higher in women [OR 1.24 (1.05–1.46), *p* = 0.01], per percent decrease in ALM/weight after controlling for age, race, height, smoking, and education (Fig. 1). After additionally controlling for android/gynoid fat, the odds of diabetes were 1.20 times higher per percent decrease in ALM/weight in men [OR 1.20 (1.04–1.37), *p* = 0.01]; an inverse association was also observed in women, albeit was not statistically significant [OR 1.08 (0.90–1.30), *p* = 0.42] (Fig. 1). After additionally controlling for physical inactivity, the odds of diabetes were 1.18 times higher per percent decrease in ALM/weight in men [OR 1.18 (1.02–1.37), *p* = 0.02]; an inverse association was also observed in women, albeit was not statistically significant [OR 1.07 (0.87–1.31), *p* = 0.54] (Fig. 1). If physical activity was instead categorized according to AHA-recommended physical activity guidelines, the results were comparable. In men, the odds of diabetes were 1.19 times higher per percent decrease in ALM/weight in men [OR 1.19 (1.01–1.39), *p* = 0.04]. In women, an inverse association was also observed, albeit was not statistically significant [OR 1.13 (0.99–1.31), *p* = 0.095).

In a sex-combined interaction model that included age, race, height, smoking, and education, the odds of diabetes were 1.26 times higher per percent decrease in ALM/weight [OR 1.26 (1.14–1.39), *p* = 0.0001] (Fig. 1). After additionally controlling for android/gynoid fat, the odds of diabetes were 1.12 times higher per percent decrease in ALM/weight [OR 1.12 (1.01–1.25), *p* = 0.04]. After additionally controlling for physical inactivity, an inverse association was also observed albeit was not statistically significant [OR 1.11 (0.98–1.25), *p* = 0.10]. If physical activity was instead categorized according to AHA recommended physical activity guidelines, the results were similar. The odds of diabetes were 1.14 times higher per percent decrease in ALM/weight [OR 1.14 (1.05–1.24), *p* = 0.003]. There was no statistically significant sex interaction in any of these models.

When the diabetes definition was changed to diabetes by self-report only so as not to introduce bias for or against subjects with missing biochemical data, effect estimates were similar, although not statistically significant, likely owing to the smaller number of diabetes cases.

### Racial differences in the association between skeletal muscle mass and diabetes prevalence

The odds of diabetes trended higher per percent decrease in ALM/weight in non-Hispanic White men after controlling for age, height, smoking, education, and android/gynoid fat [OR 1.30 (0.97–1.73), *p* = 0.08]. In non-Hispanic Black men, Hispanic men, non-Hispanic White women, non-Hispanic Black women, and Hispanic women, the association was not significant.

## Discussion

We demonstrated that less skeletal muscle mass is associated with higher odds of prevalent diabetes independent of body fat distribution in young men. For example, a 91 kg man with 28.2 kg of muscle (31% ALM/weight) had a 1.20-times higher odds of diabetes than a 91 kg man with 29.1 kg of muscle (32% ALM/weight). We add to the literature by demonstrating that this association is independent of body fat distribution, which is known to be associated with type 2 diabetes risk. However, the association was not statistically significant among women after controlling for android and gynoid adiposity. Whether low muscle mass is a causal factor in the development of type 2 diabetes, or whether it is simply a marker of higher metabolic risk, requires further investigation.

Our data are in agreement with previous sex-stratified cross-sectional studies, which reported that relatively lower muscle mass is associated with insulin resistance [20] and diabetes [7] in men, but not women. We add to the literature by demonstrating that this association between muscle mass and diabetes prevalence remains significant in men after controlling for android and gynoid adiposity. Although there is no consensus about the best biomarker to capture the association between fat distribution and type 2 diabetes risk, and we demonstrated a widespread correlation between adiposity measures, we chose the android/gynoid fat ratio for our models because it makes a distinction between android fat, which confers increased risk for type 2 diabetes, and gynoid fat, which may be protective against type 2 diabetes [17, 21, 22]. In contrast, other adiposity measures do not distinguish between different fat depots with differential risks for diabetes and whose distribution varies by sex. In contrast to the results we report in men, we found that the association between skeletal muscle mass and diabetes prevalence was not statistically significant in women after controlling for android and gynoid adiposity. We may have had more power to detect an association in men because men have more muscle mass and higher type 2 diabetes prevalence than BMI- and age-matched women [23]. Alternatively, since women have less muscle mass, but more gynoid fat, than men, perhaps muscle is a less important, while gynoid fat is a more important, depot in women to protect against diabetes risk compared to men. In addition, the exploratory race-stratified analysis was limited by small numbers, making it difficult to draw conclusions from the data. Further studies are warranted to determine whether relationships between muscle mass and diabetes are influenced by sex and race.

One strength of this cross-sectional study is that it uses a more comprehensive definition of diabetes that includes both self-reported and biochemical data, whereas previous studies investigating the association between muscle mass and prevalent diabetes did not include HbA1c and/or OGTT data [6, 7]. As expected, this resulted in a higher prevalence of diabetes than reported by the 2005–2006 National Health Interview Survey (2.4–2.7% among U.S. adults aged 18–44 years), which only defined diabetes by self-report, and thus did not include adults unaware of their diabetes diagnosis [24]. One limitation of this study is that correlation in a cross-sectional study does not prove causation. Since ALM/weight is correlated with multiple adiposity measures, the association between ALM/weight and type 2 diabetes could be subject to residual confounding by adiposity even after controlling for android/gynoid fat. Previous prospective studies in middle-aged and older adults have produced discordant results with respect to the association between muscle mass and incident diabetes [25–27], and other studies have demonstrated that type 2 diabetes is a risk factor for muscle loss with aging [28]. Since muscle and fat mass are correlated both cross-sectionally and longitudinally, interventions targeting muscle mass in the setting of randomized, placebo-controlled trials are needed in order to determine whether low muscle mass is a type 2 diabetes risk factor independent of adiposity. In addition, since we restricted our study to adults aged 20–49 years old, the results of this study may not be generalizable to older adults, who lose muscle mass with aging and have a higher prevalence of diabetes.

In summary, we demonstrate that less skeletal muscle mass independent of android and gynoid adiposity is associated with higher diabetes prevalence in young men. The association was not statistically significant in women after controlling for body fat distribution. Further study is needed to determine whether low muscle mass itself is a causal factor in the development of type 2 diabetes, or whether it is simply a marker of higher metabolic risk.

## Data Availability

All data generated and analyzed during this study are available in the following repository: National Health and Nutrition Examination Survey, https://www.cdc.gov/nchs/nhanes/index.htm.
